# Does Overseas Investment Raise Corporate Environmental Protection? Evidence from Chinese A-List Companies

**DOI:** 10.3390/ijerph19020837

**Published:** 2022-01-12

**Authors:** Quan-Jing Wang, Qiong Shen, Yong Geng, Dan-Yang Li

**Affiliations:** 1 School of Business, Zhengzhou University, 100 Science Avenue, Gaoxin District, Zhengzhou 450001, China; wqj@zzu.edu.cn (Q.-J.W.); qiongs@zzu.edu.cn (Q.S.); 2 School of Environment and Science Engineering, Shanghai Jiaotong University, 800 Dongchuan Road, Minhang District, Shanghai 200240, China; ygeng@sjtu.edu.cn; 3 School of Business, Shaanxi Normal University, 620 West Chang’an Avenue, Chang’an District, Xi’an 710069, China

**Keywords:** overseas investment, environmental protection, institutional distance, heterogeneity

## Abstract

This paper uses the relevant data of China’s listed companies from 2010 to 2018 to test the impact of overseas investment on corporate environmental protection and further examines whether the heterogeneity of the company and the heterogeneity of the host country changes this effect. The research results show that the environmental protection of overseas investment companies is significantly higher than that of other companies. The impact of overseas investment on corporate environmental protection is dynamic, and it only helps improve corporate environmental protection after three years of investment. This article is conducive to causally identifying the logical relationship between overseas investment and corporate environmental protection. The policy significance is that the government can rationally guide companies to invest abroad, and oversea investment will help enhance corporate environmental protection.

## 1. Introduction

In the process of economic transformation, the vitality of China’s market economy continues to be unleashed, which has led to rapid economic development, while major issues affecting people’s livelihoods such as environmental pollution and decline in air quality have also emerged. According to the latest “Environmental Performance Index: 2017 Report” released by Yale University, China ranks 60th out of 181 countries in the Environmental Performance Index, and although it is an improvement from 109th place in 2016, environmental pollution is still a serious problem in China (Data from: http://epi.yale.edu/sites/default/files/2017EPI_Full_Report_opt.pdf, accessed on 6 June 2021 and http://epi.yale.edu/sites/default/files/2016EPI_Full_Report_opt.pdf, accessed on 13 March 2021). The 19th CPC Central Committee’s fifth Plenary Session pointed out that we must adhere to the concept that lucid waters and lush mountains are invaluable assets, as well as the principle of respecting nature, conforming to nature, and protecting nature. It is necessary to investigate what factors can promote the environmental protection in China (Dong et al., 2012; Yang et al., 2019) [1,2]. As the main entity of the market, enterprises’ industrial activities are the primary factor that damages the environment (Hanna, 2010; Manderson and Kneller, 2012; An et al., 2021) [3,4,5] (Sulfur dioxide emissions in 2017 were 8,754,000 tons, of which industrial emissions of sulfur dioxide from enterprises accounted for 84%). Therefore, the environmental protection of enterprises also determines whether the green development policy set by the central government can effectively improve environmental quality (Madsen, 2009) [6]. Among all enterprises, overseas investment enterprise is a relatively special type, which faces dual supervision and management by the host country and China, has more access to advanced international management concepts, and needs to obtain support and resource allocation from the host country through their own actions to achieve their own interests. Therefore, overseas investment enterprises have the potential to enhance environmental protection through the three aspects above (Cole et al., 2006; Zhang et al., 2019; Wang et al., 2021) [7,8,9].

**Figure 1:**
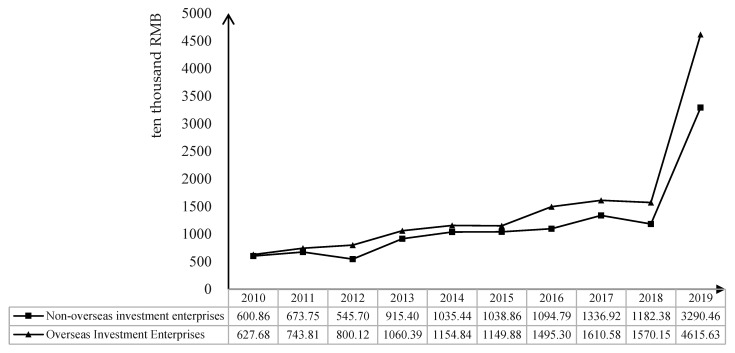
Trends in environmental expenditures of Chinese companies, 2010–2019. Data source: The annual reports of Chinese enterprises and the CSMAR database.

Firstly, under the guidance of China’s “One Belt and Road” Initiatives, the frequency of overseas investments by Chinese enterprises has gradually increased in recent years (There were 810 overseas investment events between 2010 and 2019, and the number of overseas investment events was higher than 100 each year after 2016, which implies that Chinese companies are investing more and more overseas). In order to eliminate the liability of foreignness in the host country and obtain the support of local government and the recognition of surrounding residents, so as to obtain more political and productive resources, overseas investment enterprises have an incentive to produce and operate in an environmentally friendly way to reduce the environmental damage caused by their actions (Spatareanu, 2007; Marano et al., 2016) [10,11]. Secondly, the production behavior of overseas investment enterprises needs to comply with the requirements of the host country, China, and even the international system. For legitimacy motives, overseas investment enterprises usually raise their own environmental protection and reduce the negative externalities of their production behavior (Cheung, 2015) [12]. In addition, overseas investment firms can take advantage of the reverse spillover effect (Marano and Kostova, 2016) [13] to learn the advanced management concepts and production methods from the host country, which can help to improve the environmental protection from the perspective of management’s moral motivation. Finally, overseas investment enterprises not only face the constraints of relevant environmental protection laws and regulations in China but also bear the supervision of their environmental behavior by the host country, i.e., overseas investment enterprises are under the surveillance pressure of the dual environmental protection system from the host country and the home country (Wang et al., 2013; Gorodnichenko et al., 2014) [14,15]. Furthermore, oversea investment provides the enterprises more access to use the environmentally materials, which can do some good to improve the environmental protection (Pomares et al., 2018; Bautista et al., 2019) [16,17]. From this perspective, overseas investment has improved the environmental protection of enterprises (Zomorrodi and Zhou, 2017) [18]. Figure 1 depicts the trends in environmental protection expenditure of Chinese overseas investment and non-overseas investment firms from 2010 to 2019 (Since the 1972 United Nations Conference on the Human Environment in Stockholm, countries around the world have gradually reached a consensus to abandon unsustainable development models and achieve sustainable development, which has made the protection of the environment a global topic) (The environmental expenditures of the corresponding types of enterprises are indicated by calculating the average value of environmental expenditures of overseas-invested enterprises and non-overseas-invested enterprises for each year). Firstly, between 2010 and 2018, the environmental protection expenditure of two types of companies only declined in 2012 and 2018, which showed an upward trend as a whole, and a sharp increase in 2019. Secondly, between the two types of enterprises, the environmental protection expenditure of overseas investment enterprises is higher than that of non-overseas investment enterprises every year, which, to some extent, verifies the above inference of this paper.

The economic and social impacts of overseas investments by Chinese enterprises are multifaceted. In recent years, academics have gradually conducted in-depth studies on the economic and social impacts generated by overseas investments, for example, some studies have found that Chinese enterprises’ overseas investments regulate corporate behavior and improve corporate social responsibility, technological innovation, and additive rates to a certain extent (Araya, 2012; Aung, 2021) [19,20]. However, as of yet, no literature has been found to analyze and explore the impact of overseas investment on firms’ environmental protection. The increasing number of Chinese overseas investment enterprises provides an opportunity to study this issue. Analyzing and exploring the impact of overseas investment on corporate environmental protection from the perspective of institutional distance can not only further expand and enrich relevant studies on overseas investment and corporate behavior theoretically but also provide a new perspective and theoretical basis for relevant government departments to formulate relevant policies to enhance the green and sustainable development of enterprises.

Based on the above understanding, this paper uses the relevant data of China’s listed companies from 2010 to 2018, the two-way panel fixed effects model and the PSM-DID measurement method to empirically examine the impact of overseas investment on corporate environmental protection and its dynamics. The findings suggest that overseas investment improves corporate environmental protection and the impact of overseas investment on corporate environmental protection is dynamic in nature, with corporate environmental protection improving after three years following overseas investment.

Compared with the existing literature, the main contributions of this paper are mainly reflected in the following aspects: it expands the literature on how overseas investment affects firms, which contributes to a more comprehensive and deeper understanding of the impact of overseas investment on the parent company. Unlike many studies that take parent company’s corporate performance, corporate innovation, and production capacity as their entry points, this study focuses on corporate environmental protection, which is at the core of corporate green sustainability, and confirms the positive impact of overseas investment on corporate environmental protection.

The remainder of the paper is organized as follows: The second part is the theoretical mechanism that explains how overseas investment influences corporate environmental protection. The third part is the research design, which explains the variables, data sources, and model settings. The fourth part presents the empirical results, which show the impact of overseas investment on the environmental protection of enterprises. The fifth part is the conclusion of the study and policy recommendations.

## 2. Theoretical Analysis and Hypothesis Formulation

### Foundational Theoretical Hypotheses

There are few studies which study the impact of overseas investment on corporate environmental protection, and only some of them have addressed the impact of overseas investment on the environment in the process of research. Wang (2017) [21] used data that related to overseas investment of Chinese listed companies and CSR on Hexun.com (accessed on 13 May 2021), as well as the PSM-DID method, to empirically test the impact of overseas investment on CSR and then pointed out that overseas investment urges companies to take more environmental responsibility. From the side, it shows that overseas investment is conducive to improving corporate environmental protection.

We believe that overseas investment will raise corporate awareness of environmental protection through the following channels. First of all, overseas investment affects corporate environmental protection through reverse spillover and learning effects. Overseas enterprises can invest in the host country (Morck et al., 2008; Abdo et al., 2020) [22,23] and use technology diffusion, information exchange, and imitation learning, etc. (Liu et al., 2018) [24], to absorb the host country’s advanced technological knowledge, corporate culture, and management experience so as to obtain positive reverse spillover effects, including the green technology of host country enterprises and their environmental protection concepts, which improve overseas investment enterprises’ attention to the environment and thus help to enhance their environmental protection awareness (Ning and Wang, 2018) [25]. Secondly, overseas investment enhances corporate awareness of environmental protection through the “spotlight” effect. Overseas investment is a hot event, which raises the attention of the government, the public, and the media to the enterprise (Sapkota and Bastola, 2017; Bhujabal et al., 2021) [26,27]. In the “spotlight” effect brought by overseas investment, the pollutant emissions and the environmental damage caused by their production activities are more likely to be exposed, which to a certain extent motivates enterprises to adopt more environmentally friendly ways to produce or allocate more investment to eliminate pollution caused by their own activities, which enhances the environmental protection of enterprises (Bildirici and Gokmenoglu, 2020) [28]. Thirdly, overseas investment motivated by legitimacy raises corporate environmental protection. In order to eliminate the disadvantage of being an outsider in the host country and to obtain the right to use resources and government support there, as well as to improve the recognition of the enterprise by the surrounding people and the market, overseas investment enterprises will pay more attention to reducing the negative externality of their production activities and minimizing the damage to the host country’s environment by investing more in environmental protection in the context of the world’s concern for environmental issues (Blanco et al., 2013; Albulescu et al., 2019) [29,30]. Along with the transformation of overseas subsidiaries, this behavior will eventually influence the operations of the parent company and raise its awareness of environmental protection (Demena and Afesorgbor, 2020; Hao et al., 2020) [31,32]. Finally, overseas investment enhances the environmental protection awareness of enterprises through the dual institutional pressure from the host country and China. Compared with ordinary enterprises, overseas investment enterprises are not only bound by the Chinese system but are also influenced by the host country system. The dual system pressure means that overseas investment enterprises have stricter standards of behavior, including environmental protection regulations. The higher environmental protection standards, the greater environmental protection (Rafindadi et al., 2018; Sabir et al., 2020) [33,34].

In addition, after making overseas investments, enterprises need a certain amount of time to learn and absorb the concept before they can effectively improve their environmental protection, whether through the reverse spillover effect or the learning effect to actively change their own production methods or due to the dual institutional pressure from the host country and China to passively change their production mode. Based on data related to Chinese listed firms, Wang and Liu (2019) show that overseas investment has a positive impact on corporate environmental responsibility, and this impact is dynamic and time-lagged. Based on this, this paper proposes the following hypotheses:

**Hypothesis** **H1.** *Overseas investment improves corporate environmental protection.*

**Hypothesis** **H2.** *The positive impact of overseas investment on corporate environmental protection is dynamic in nature.*

## 3. Variables, Data, and Methodology

### 3.1. Variables Selection and Data Sources

Corporate environmental protection (Envirpro): Drawing on the approach of Zeng et al. (2010) [35] as well as Jiang and Akbar (2018) [36], this paper uses the proportion of the enterprise’s environmental protection expenditure in the year to the enterprise’s asset size to measure its environmental protection, which is expressed as Enviropro. The more spending on environmental protection, the more environmentally conscious and the greater Enviropro.

Overseas investment (Oversea): Drawing on the study by Zhou et al. (2002) [37] and Hoang et al. (2021) [38], the dummy variable for overseas investment is set according to whether a firm makes overseas M&A, which is denoted by Oversea. Specifically, when a firm makes an overseas investment in a given year from 2010–2018, the firm is classified as an overseas investing firm for that year and subsequent years, and Oversea = 1, otherwise, Oversea = 0.

Drawing on existing studies, the control variables in this paper are selected as follows: equity concentration (CR), number of directors (DSRS), board independence (DDRS), separation of powers ratio (LQFL), dual role (LZHY), return on net assets (ROE), financial leverage (CWGG), enterprise risk (ZHGG), current ratio (LDR), firm size (Size), firm attributes (Nature), industry concentration (HHI), industry return on total assets (INDROA), and industry net gearing ratio (INDLEV). The specific definitions of each variable are given in Table 1 and are not repeated here.

### 3.2. Data

The research samples of this paper are A-share listed companies. Environmental expenditures are hand-searched from corporate annual reports. Corporate overseas investment data are obtained from the CSMAR database. Corporate financial data are obtained from Wind as well as the CSMAR database. The time frame of this paper is set to 2010 to 2018. It involves 810 overseas investment events. The stock codes and years of listed companies are used as the subject, and then all data are matched together to form the original sample of this paper. To enhance the data quality and credibility of the conclusions in this paper, the original samples are further clarified according to the following principles: (1) exclude ST or *ST enterprises; (2) exclude the financial industry; (3) exclude the sample with missing data of enterprise environmental protection expenditure; (4) removing out the upper and lower 1% for the variables with extreme values; (5) exclude the sample observations with a duration of less than three years.

Table 2 shows the descriptive statistics of the variables in the text. Firstly, in terms of environmental protection, the environmental expenditure variable (Envirpro) has a mean value of 14.582, with minimum and maximum values of 0 and 20.150, respectively, and a standard deviation of 1.923, implying that there is some variation in the environmental expenditure among different firms. The mean value of the overseas investment variable (Oversea) is 0.1, implying that the share of overseas investing firms is about 10% during the period 2010 to 2018. The statistical distribution of other variables is shown in Table 2, and we will not expand the analysis here.

In addition, the last column of Table 2 gives the correlation coefficient between environmental protection and other variables, from which it can be seen that the correlation coefficient between Envirpro and Oversea is 0.07, which passes the significance test at the 1% level, implying that overseas investment helps to increase the environmental protection of firms.

### 3.3. Estimating Methods

#### 3.3.1. Full Sample Regression

Given that this paper uses panel data, the Hausman test is first used to filter out the appropriate regression method from mixed regression, panel random effects model, and panel fixed effects. The results indicate that the fixed effect is more appropriate (Hausmann’s test results are not reported and are available from the authors upon request). The econometric model is set as follows:

(1) $${Envirpro}_{it}=C_{0}+\alpha_{0}{Oversea}_{it}+\beta_{0}’X+\mu_{i}+\mu_{t}+\varepsilon_{it}$$

Here, *Envirpro* is the explained variable, which represents the environmental protection of enterprises; Oversea is the explanatory variable, which represents the dummy variable of overseas investment; X is the other control variables; and *β_i_* represents the coefficients of the corresponding control variables. To control of the effect of year on corporate environmental protection, this paper uses a two-way fixed-effects model, in which a time variable is included, $\mu_{i}$ represents individual fixed effects. *μ_t_* represents year fixed effects. *ε_it_* is the residual term.

#### 3.3.2. PSM-DID Regression

To avoid sample selection bias and endogeneity due to bidirectional causality between variables, this paper using the propensity score matching method (PSM) to firstly find a control group matched with overseas investment firms, which can construct a counterfactual sample to address the bias deficiency in sample selection. Secondly, a difference-in-differences model (DID) is used to estimate the true treatment effect of overseas investment on the environmental protection of the investee firms.

(1) Propensity Score Matching Method (PSM)

The full sample A is divided into two groups. One is the treatment group: If a firm has made overseas investment during 2011–2018, the firm is classified as the treatment group and is denoted as T. The other group is the control group, i.e., the firm that has not made overseas investment, and is denoted as C; then, A = {C, T}. The matching is performed by finding firms in C that have a similar probability of conducting the experiment as the overseas investing firms in T, which can solve the sample selection problem.

Assume that the probability of a firm investing abroad is:

(2) $$P=\mathrm{Pr}\{A=T\}=\phi \{X_{i,t-1}\}$$

Here, the probability of a firm investing overseas is *P*; *X* is the matching variable, which is the characteristic variable that indicates the influence of the firm being invested abroad and the firm’s environmental protection, which is used to ensure that the negligibility assumption is satisfied. Drawing on existing studies, equity concentration (CR), number of directors (DSRS), board independence (DDRS), separation of powers rate (LQFL), dual positioning (LZHY), return on net assets (ROE), financial leverage (CWGG), corporate risk (ZHGG), current ratio (LDR), firm size (Size), firm attributes (Nature), industry concentration (HHI), industry return on total assets (INDROA), and industry net gearing (INDLEV) are used as matching variables. The matched variables are all lagged by one period. Based on the measurement results, firms with similar probability of overseas investment to those in the treatment group are selected from the control group as the control sample, denoted by Cp. In the PSM method, the K-nearest neighbor matching method is selected in this paper, and the one-to-one nearest neighbor matching method without put-back is chosen.

(2) Difference-in-differences model (DID)

After propensity score matching, another set of samples Ap = {Cp, T} was obtained. If the sample is the treatment group, then Treat = 1; otherwise, Treat = 0. After performing PSM, the two samples have nearly the same probability of investing overseas, and a smoothness test shows that there are no significant individual differences in the samples. As a result, either of them can be treated as a natural experiment.

For the econometric model, a difference-in-differences regression can be performed using the following model:

(3) $${Envirpro}_{it}=C_{0}+\varphi_{1}{Before1}_{it}+\varphi_{2}{Before2}_{it}+\lambda_{0}{Treat}_{i}+\lambda_{1}{Time}_{it}+\theta_{0}{Treat}_{i}\times {Time}_{it}+\beta_{0}’X+\mu_{t}+\varepsilon_{it}$$

Here, *Treat* represents the sample group to which the firm belongs, i.e., it measures the mean difference between the treatment and control groups. According to this setting, the coefficient of $Treat_{i}\times Time\;\theta_{i}$, i.e., the effect of overseas investment on firms’ environmental protection, is the core variable of interest in this paper. The time fixed effect is $\mu_{t}$, and given that the matched samples do not differ significantly between individuals, individual effects are no longer controlled for. The random error term is *ε*_it_; in line with the theoretical underpinnings of the difference-in-differences approach, Before1 and Before2 are the antecedent variables for overseas investment and are used to test the parallel trend assumption.

## 4. Estimating Results

### 4.1. T-Test between Groups

Table 3 presents the results of the t-test between groups. Among non-foreign invested firms, the mean value of corporate environmental protection expenditure is 14.542, while among overseas invested firms, the mean value of corporate environmental protection expenditure is 14.946. In terms of corporate environmental protection expenditure, non-foreign invested firms are 0.404 lower than overseas invested firms and this difference passes the significance test at the 1% level. The between-group *t*-test likewise indicates that overseas investment helps to improve corporate environmental protection awareness.

### 4.2. Two-Way Fixed Effects Results

Based on the panel two-way fixed effects model, Table 4 reports the estimation results, i.e., the impact of overseas investment on corporate environmental expenditures. The explained variables in column (1)–(5) are the level of corporate environmental expenditure (Envirpro), and the explanatory variables are overseas investment (Oversea); Equations (1)–(3) add the time fixed effect, corporate governance variable, corporate financial indicators, firm characteristics, and industry control variables to the regression model in that order. Controlling for time fixed effect, the coefficient of Oversea in column (1) is 0.311, which is significantly positive at the 5% level. This result indicates that overseas investment significantly increases environmental expenditure, i.e., overseas investments help to increase firms’ environmental protection. The coefficients of Oversea in column (2)–(5) are positive and pass the significance test at least at the 10% level, verifying the positive impact of direct overseas investment on corporate environmental protection expenditure again.

### 4.3. Robustness Examination

To verify the reliability of the results of the baseline regressions in this paper, this section further tests the impact of overseas investment on firms’ environmental investment using the following two methods. Firstly, the explained variables are transformed. Corporate green invention patents need to consume a large amount of environmental protection R&D investment, and more green patents imply higher environmental protection expenditure. Therefore, this section uses green invention patents as a proxy indicator for environmental protection input to empirically test the impact of overseas investment on corporate environmental protection input. Secondly, to avoid sample selection bias and endogeneity caused by ignoring other unobservable firm characteristics, this paper firstly uses the propensity score matching method (PSM) to find a control group matching with overseas investment firms to construct a counterfactual sample which can address the bias deficiency in sample selection. Secondly, the difference-in- difference model (DID) is used to estimate the true treatment effect of overseas investment on firms’ environmental protection.

#### 4.3.1. Changing the Explained Variables

Since innovation takes some time, this section uses the one-period-ahead and two-period-ahead green invention patents as the explained variables to analyze the impact of overseas investment on enterprises’ environmental protection input, and the regression results are shown in Table 5. The coefficient of Oversea in column (1) is 0.251, which passes the significance test at the 1% level and has a positive sign, implying that overseas investment significantly improves their green technology innovation, i.e., overseas investment helps to improve the environmental protection of the firms. After adding other control variables that affect the environmental protection of enterprises, the coefficient of Oversea in column (2) is 0.266, which is significantly positive at the 1% level, meaning that overseas investment positively improves the green technology innovation of enterprises. After transforming the explained variables into the two-period-ahead green technological innovation, the coefficient of Oversea in column (3) is 0.232, which is significantly greater than 0 at the 1% level, verifying the positive effect of overseas investment on corporate green technological innovation; the regression results of column (4) in which other control variables are added also support this conclusion.

#### 4.3.2. PSM-DID Estimation Results

In this paper, a year-by-year and one-to-one nearest neighbor matching method without put-back is selected for the full sample. To verify that the matching results are reliable, a balance test is performed on the propensity score matching for each year. Since the tables are relatively similar in structure, they are not reported. In this paper, as an example, we only report the results of the balance test of propensity score matching using the 2010 matching results.

According to the balance test results in Table 6, the standard deviations of all matched variables are reduced to some extent after the matching treatment. Before matching, there are significant differences between the control and treatment groups in terms of separation of powers ratio (LQFL), dual employment (LZHY), financial leverage (CWGG), combined leverage (ZHGG), firm size (Size), firm attributes (Nature), industry concentration (HHI), and so on. After matching, these variables do not differ significantly between the treatment and control groups, ensuring the credibility of the subsequent PSM-DID results in this paper.

Based on the obtained PSM1 to 1 sample, this section uses the difference-in-difference method to estimate the effect of overseas investment on firms’ environmental protection, and the results are presented in regression (i) in Table 7. Firstly, the estimation results of the one-period-ahead and two-period-ahead variables (Before1 and Before2) for overseas investment are used to analyze the parallel trend check of the DID regression. The coefficients of both Before1 and Before2 in Equation (1) do not pass the significance test at the 10% level, indicating that there is no significant difference in corporate environmental protection between the two sample groups of firms prior to overseas investment, which satisfy the parallel trend assumption. Next, the coefficient of Treat*Time in Equation (1) is 0.449, which is significantly positive at the 5% level, implying that the environmental protection of overseas invested firms is higher compared to non-overseas invested firms. After transforming the matching method to a 1-to-3 nearest neighbor matching method with put-back, the estimation is developed based on the new matched sample and the results are presented in Equation (2) in regression (i) in Table 7. The results in Equation (2) continue to support this finding.

#### 4.3.3. Results of Dynamic Effect Estimates

Based on the PSM1 to 1 and PSM1 to 3 samples, this section further tests the dynamic effect of overseas investment on firms’ environmental protection, and the regression results are presented in regression (ii) in Table 7. The coefficients of the period-ahead variables (Before1 and Before2) in Equation (3) are not significant at the 10% level, indicating that the parallel trend assumption is satisfied. The coefficient of After_0 in Equation (3) is −0.286, which is not significant at the 10% level, implying that overseas investment does not change the environmental protection of firms in the year of overseas investment. The coefficient of After_1 is 0.110, which does not pass the significance test at the 10% level, indicating that the effect of overseas investment on the environmental protection of firms is not significant in the first year after overseas investment. The coefficient of After_2 is 0.314, which is not significant at the 10% level, implying that overseas investment did not change the environmental protection of enterprises in the second year after overseas investment. The coefficient of After_3 is 0.645, which is significantly positive at the 5% level, implying that in the third year after overseas investment, overseas investment significantly increased the environmental protection of enterprises. In addition, the signs of the coefficients of After_4–After_5 are all positive and pass the significance test, at least at the 5% level. The results of Equation (3) indicate the dynamic nature of the impact of overseas investment on corporate environmental protection. In the year of overseas investment and the following two years, the investment does not change the environmental protection of the companies. However, it is only after three years of overseas investment that overseas investment significantly improves corporate environmental protection. The results based on PSM1 to 3 in Equation (3) also support this finding.

## 5. Conclusions and Policy Implications

### 5.1. Research Conclusions

Based on the theoretical analysis, this paper empirically tests the impact of overseas investment on parent companies’ environmental protection by using data related to A-share listed companies in China and overseas investment from 2010–2018, as well as using a combination of two-way panel fixed-effects model, fixed-effects count model and PSM-DID model, and further analyzes the dynamics of this impact. After determining the effect of overseas investment on corporate environmental protection, the difference of the effect of overseas investment on corporate environmental protection is discussed in terms of both parent company heterogeneity and host country heterogeneity across different firms.

This paper finds that, firstly, overseas investment helps to improve corporate environmental protection in terms of both environmental spending and green technology innovation. Secondly, the impact of overseas investment on firms’ environmental protection is dynamic in nature. In the year of the overseas investment and the two years afterwards, the environmental protection of enterprises did not change, while three years after the overseas investment, the environmental protection of enterprises improved.

### 5.2. Policy Recommendations

Accordingly, this paper puts forward the following policy recommendations: Firstly, in the complicated international situation, the government should focus on improving relevant regulations and international safeguards to reduce the risks of enterprises’ foreign investment in order to guide more enterprises to go abroad and regulate their overseas investment behavior. Through the reverse spillover effect, the learning effect of enterprises in the host country and the institutional constraints on the behavior of enterprises, firms’ awareness of environmental protection will be improved. Secondly, the impact of overseas investment on enterprises’ environmental protection is not immediate, which takes some time. Therefore, the government should formulate corresponding preferential policies to encourage enterprises to make more strategic long-term foreign investment rather than short-term profitable foreign investment. Only after long-term learning and change in production methods can corporate environmental investment be improved.

## Figures and Tables

**Table 1:** Variable definitions.

Variable Type	Variable Name	Variable Symbol	Variable Definition
Explained variables	Corporate environmental expenditure	Envirpro	Environmental expenditure of the enterprise for the year as a percentage of the enterprise’s asset size
Explanatory variables	Overseas direct investment	Oversea	If a firm makes an overseas investment in a given year, the firm is defined as an overseas investment firm for that year and subsequent years with Oversea = 1; otherwise, Oversea = 0.
Control variables	Enterprise level		
	Concentration of shareholding	CR	Sum of the number of shares held by the top ten shareholders for the year/total number of shares
	Number of Directors	DSRS	Number of all directors on the board at the end of the year
	Number of Independent directors	DDRS	Number of independent directors at the end of the year
	Separation of powers rate	LQFL	Extent of separation of ownership and operation of the enterprise
	Dual employment	LZHY	Whether the chairman and general manager are the same person, if yes, LZHY = 1, otherwise, LZHY = 0
	Return on net assets (ROAN)	ROE	Total EBITDA for the year/Average total net assets
	Financial leverage	CWGG	Total liabilities/total assets of the enterprise at the end of the year
	Enterprise risk	ZHGG	Rate of change in profit per ordinary share of the enterprise for the year/rate of change in sales volume
	Current ratio	LDR	Current assets/current liabilities at the end of the year
	Business Size	Size	Natural logarithm of the total assets of the enterprise at the end of the year
	Business Attributes	Nature	Generate dummy variables based on the nature of the actual controller of the business
	Industry level		
	Industry concentration	HHI	Based on the dichotomous industry codes disclosed by the CSRC, the Herfindahl index is calculated year by year using the operating revenues of companies in the industry to measure industry concentration, which is used as an inverse indicator of industry concentration and is denoted as HHI
	Industry Total Return on Assets	INDROA	Based on the dichotomous industry codes disclosed by the CSRC, the compensation rate of total assets of companies in the industry is weighted year by year to take the average value, using total assets as the weighting
	Total industry gearing ratio	INDLEV	Based on the dichotomous industry codes disclosed by the CSRC, the total gearing ratio of enterprises in the industry is weighted year by year to take the average value, using total assets as the weighting

**Table 2:** Summary of descriptive statistics.

Variable	N	Mean	SD	Min	Median	Max	Correlation Coefficient
Envirpro	4069	14.58	1.92	0.00	14.66	20.15	
Oversea	4069	0.10	0.30	0.00	0.00	1.00	0.07 ***
CR	4012	57.21	15.73	22.41	57.59	94.44	0.07 ***
DSRS	4031	8.89	1.83	4.00	9.00	19.00	0.16 ***
DDRS	4031	3.24	0.65	0.00	3.00	8.00	0.17 ***
LQFL	3855	5.58	8.31	−35.09	0.00	40.18	0.10 ***
LZHY	3986	1.79	0.41	1.00	2.00	2.00	0.09 ***
ROE	4042	−0.01	1.90	−74.76	0.06	26.68	0.03
CWGG	3991	1.56	1.39	−0.73	1.12	7.59	0.01
ZHGG	3991	2.83	3.60	−2.50	1.69	18.64	0.01
LDR	4042	2.08	2.94	0.00	1.34	68.97	−0.20 ***
Size	3955	22.22	1.18	18.64	22.14	27.67	0.53 ***
Nature	3728	0.52	0.50	0.00	1.00	1.00	0.18 ***
HHI	4069	0.10	0.10	0.02	0.07	1.00	0.02
Indroa	4069	0.04	0.05	−0.23	0.04	0.87	−0.08 ***
Indlev	4069	0.45	0.11	0.17	0.43	1.46	0.09 ***

Notes: *** indicates statistical significance at the 1% level.

**Table 3:** *T*-test between groups.

Variable	N (0)	Mean (0)	N (1)	Mean (1)	Mean-Diff	*t*
Envirpro	3664	14.54	405	14.95	−0.40 ***	−4.02

Notes: *** indicates statistical significance at the 1% level.

**Table 4:** Mixed sample regression results.

	(1)	(2)	(3)	(4)	(5)
Oversea	0.31 **	0.32 **	0.31 **	0.23 *	0.23 *
	(2.42)	(2.43)	(2.38)	(1.72)	(1.77)
CR		0.01	0.01	−0.01	−0.01
		(0.05)	(0.38)	(−1.12)	(−1.19)
DSRS		0.01	−0.01	−0.01	0.01
		(0.02)	(−0.19)	(−0.00)	(0.04)
DDRS		0.02	0.03	0.01	−0.01
		(0.21)	(0.38)	(0.02)	(−0.00)
LQFL		0.01	0.01	0.01	−0.01
		(0.77)	(0.73)	(0.01)	(−0.00)
LZHY		−0.06	−0.09	−0.09	−0.10
		(−0.76)	(−1.16)	(−1.19)	(−1.20)
ROE			0.01	−0.01	−0.01
			(0.79)	(−0.38)	(−0.38)
CWGG			−0.01	−0.01	−0.01
			(−1.23)	(−0.96)	(−0.96)
ZHGG			0.01	0.01	0.01
			(1.22)	(0.97)	(0.97)
LDR			−0.01 **	−0.01	−0.01
			(−2.22)	(−1.03)	(−1.05)
Size				0.48 ***	0.48 ***
				(5.81)	(5.76)
Nature				−0.38	−0.38
				(−1.32)	(−1.32)
HHI					0.59
					(1.42)
Indroa					−0.35
					(−1.19)
Indlev					0.05
					(0.11)
Year	control	control	control	control	control
Individual	control	control	control	control	control
Cons	14.37 ***	14.54 ***	14.58 ***	4.25 **	4.22 **
	(231.85)	(38.44)	(37.95)	(2.29)	(2.23)
N	4069	3798	3753	3449	3449
R2	0.02	0.02	0.04	0.26	0.25
F	4.36	3.17	3.10	4.08	3.65

Notes: ***, **, and * indicate statistical significance at the 1%, 5%, and 10% levels, respectively. t-statistics are in parenthesis.

**Table 5:** Robustness Test—Transforming the explained variables.

	(i) GI _t + 1_		(ii) GI _t + 2_	
	(1)	(2)	(3)	(4)
Oversea	0.25 ***	0.27 ***	0.23 ***	0.21 **
	(3.21)	(2.65)	(2.92)	(2.01)
CR		0.01		0.01
		(0.04)		(0.85)
DSRS		−0.01		0.10 ***
		(−0.40)		(3.01)
DDRS		0.15 **		−0.17 **
		(2.14)		(−2.31)
LQFL		−0.02 ***		−0.02 ***
		(−3.89)		(−3.75)
LZHY		0.18 *		0.06
		(1.85)		(0.56)
ROE		−0.02		−0.05
		(−0.59)		(−1.31)
CWGG		−0.01		0.03
		(−0.82)		(1.46)
ZHGG		0.01		−0.01
		(0.85)		(−1.50)
LDR		−0.01		−0.02 **
		(−0.91)		(−1.96)
Size		0.52 ***		0.54 ***
		(8.36)		(7.19)
Nature		−0.65 **		−0.31
		(−2.14)		(−0.93)
HHI		−0.66		−1.14 **
		(−1.47)		(−2.39)
Indroa		0.12		−0.27
		(0.58)		(−1.50)
Indlev		1.06 ***		−0.06
		(4.19)		(−0.24)
Year	control	control	control	control
Individual	control	control	control	control
N	4422	3752	3666	3141
Wald	1230.34	808.00	679.46	598.08
P	0.01	0.01	0.01	0.01

Notes: ***, **, and * indicate statistical significance at the 1%, 5%, and 10% levels, respectively. Z-statistics are in parenthesis.

**Table 6:** Propensity score matching balance test, 2010.

Variable Name		Average Value		Standard Deviation (%)	Reduction in Standard Deviation (%)	*t*-Statistic	*t*-Test P > T
		Processing Group	Control Group				
CR	pre-match	59.34	62.18	−17.10		−0.82	0.42
	post-match	59.34	58.81	3.20	81.30	0.19	0.85
DSRS	pre-match	9.36	9.04	17.10		1.15	0.25
	post-match	9.36	9.19	8.90	47.60	0.43	0.67
DDRS	pre-match	3.29	3.28	1.50		0.10	0.92
	post-match	3.29	3.29	0.00	100.00	0.00	1.00
LQFL	pre-match	8.21	5.09	36.00		2.50	0.01
	post-match	8.21	10.17	−22.60	37.10	−0.90	0.37
LZHY	pre-match	1.91	1.77	36.70		2.04	0.04
	post-match	1.91	1.88	6.50	82.20	0.35	0.73
ROE	pre-match	0.11	0.08	8.70		0.40	0.69
	post-match	0.11	0.11	1.40	83.40	0.32	0.75
CWGG	pre-match	0.75	1.29	−24.50		−2.94	0.01
	post-match	0.75	0.91	−7.20	70.50	−0.31	0.76
ZHGG	pre-match	0.94	2.04	−24.70		−2.15	0.03
	post-match	0.94	1.07	−2.90	88.20	−0.12	0.90
LDR	pre-match	3.36	4.70	−23.30		−0.86	0.39
	post-match	3.36	3.64	−4.80	79.40	−0.26	0.80
Size	pre-match	22.02	21.59	37.70		2.56	0.01
	post-match	22.02	21.98	3.70	90.10	0.16	0.87
Nature	pre-match	0.38	0.51	−26.10		−1.65	0.10
	post-match	0.38	0.36	4.80	81.60	0.22	0.82
HHI	pre-match	0.12	0.08	29.70		1.80	0.08
	post-match	0.12	0.12	−1.80	94.00	−0.11	0.91
Indroa	pre-match	0.05	0.06	−9.20		−0.38	0.70
	post-match	0.05	0.05	6.20	32.60	0.48	0.63
Indlev	pre-match	0.52	0.48	18.60		0.99	0.32
	post-match	0.52	0.52	−0.90	95.20	−0.06	0.95

**Table 7:** Robustness Test-PSM-DID.

	(i) Static Effect		(ii) Dynamic Effect	
	(1) PSM 1:1	(2) PSM 1:3	(3) PSM 1:1	(4) PSM 1:3
Before1	−0.20	−0.17	−0.16	−0.15
	(−1.15)	(−1.25)	(−0.95)	(−1.10)
Before2	−0.07	−0.16	−0.04	−0.15
	(−0.39)	(−1.20)	(−0.24)	(−1.10)
Treat	−0.06	0.01	0.06	0.07
	(−0.42)	(0.03)	(0.45)	(0.63)
Time	−0.17	0.02	−0.01	0.10
	(−0.90)	(0.17)	(−0.04)	(0.76)
Treat*Time	0.45 **	0.36 **		
	(2.16)	(2.14)		
After_0			−0.29	−0.25
			(−1.07)	(−1.04)
After_1			0.11	0.06
			(0.43)	(0.27)
After_2			0.31	0.26
			(1.15)	(1.03)
After_3			0.65 **	0.69 **
			(2.26)	(2.46)
After_4			0.93 ***	1.10 ***
			(2.88)	(3.15)
After_5			0.87 **	1.15 ***
			(2.41)	(2.87)
Control variables/time	control	control	control	control
N	947	1539	947	1539
R2	0.29	0.33	0.30	0.34
F	43.13	52.80	36.97	45.38

Notes: ***, and ** indicate statistical significance at the 1%, and 5%, levels, respectively. *t*-statistics are in parenthesis.

## Data Availability

The data used to support the findings of this study are available from the corresponding author upon request.
